# Draft Genome Sequence of *Pseudomonas* sp. BDAL1 Reconstructed from a Bakken Shale Hydraulic Fracturing-Produced Water Storage Tank Metagenome

**DOI:** 10.1128/genomeA.00033-17

**Published:** 2017-03-16

**Authors:** Daniel Lipus, Daniel Ross, Kyle Bibby, Djuna Gulliver

**Affiliations:** a Department of Civil and Environmental Engineering, University of Pittsburgh, Pittsburgh, Pennsylvania, USA; b Department of Computational and Systems Biology, University of Pittsburgh, Pittsburgh, Pennsylvania, USA; c National Energy Technology Laboratory (NETL), Pittsburgh, Pennsylvania, USA; d AECOM, Pittsburgh, Pennsylvania, USA

## Abstract

We report the 5,425,832 bp draft genome of *Pseudomonas* sp. strain BDAL1, recovered from a Bakken shale hydraulic fracturing-produced water tank metagenome. Genome annotation revealed several key biofilm formation genes and osmotic stress response mechanisms necessary for survival in hydraulic fracturing-produced water.

## GENOME ANNOUNCEMENT

Microbial activity associated with hydraulic fracturing-produced water and subsequently the hydraulic fracturing infrastructure is considered a significant operational concern due to the potential for corrosion, souring, and biofouling (1–4). Several studies have recently investigated the microbial community structure associated with hydraulic fracturing-produced water and hydraulic fracturing facilities (1, 5, 6). These investigations have provided interesting insights into the taxonomy of microbial populations associated with these environments. However, little is known about the functional potential and metabolic ability of these microorganisms that may result in interference with hydraulic fracturing operations.

Here, we present the draft genome of *Pseudomonas* sp. strain BDAL1, assembled from the metagenome of Bakken shale hydraulic fracturing-produced water, sampled from a produced water storage tank. Sequencing libraries were prepared using Illumina Nextera XT and sequenced using Illumina MiSeq technology (Illumina, San Diego, CA). Sequencing reads were quality trimmed (Q30) and *de novo* assembled into contigs using CLC-Genomics-Workbench version 8.5.1 (http://www.clcbio.com/products/clc-genomics-workbench) and SPAdes version 3.5.1 (7). Assembled contigs were grouped into genome bins with Vizbin (8) and taxonomy assessed with PhyloPythia (9). Metagenomic reads were mapped against binned contigs and reassembled using SPAdes.

The final draft genome contained 128 contigs of 5000 bp to 243,670 bp in length and an *N*_50_ of 63,183 bp. The total genome size was 5,425,832 bp with a mean G+C content of 58.4% and had an average of 19-fold coverage. Draft genome completeness and contamination were estimated using 833 *Pseudomonas* marker genes in CheckM (10). The final draft genome was found to be 97.4% complete and contain 0.4% contamination.

The draft genome was annotated by Rapid Annotations using Subsystems Technology (RAST) (11, 12) revealing 4,998 gene coding sequences (CDs) and 51 RNA sequences (48 tRNA, 16S, 23S, and 5S rRNA). Phylogenetic analysis of the recovered 16S rRNA gene sequence using BLASTn suggested that *Pseudomonas* sp. strain BDAL1 is closely related to *Pseudomonas syringae* pv. phaseolicola 1448A. Whole-genome alignment using an average nucleotide identity calculator confirmed the close phylogenetic relationship (99.9% nucleotide identity) (13, 14). RAST (11, 12) annotation allowed the discovery of genes participating in biofilm formation processes. These included members of the *alg* family involved in alginate metabolism and the levan production gene levansucrase (15, 16). Furthermore, multiple genes of the polysaccharide synthase locus *psl* and the glycerol transferase *glt2*, involved in exopolysaccharide (EPS) production were identified (16, 17). Annotation also allowed the identification of the osmotic stress response genes *ProX*, *ProU*, and the *Trk* complex suggesting the potential for potassium ion uptake and osmoprotectant accumulation.

The recovery and analysis of the *Pseudomonas* sp. BDAL1 draft genome revealed a high number of biofilm formation genes, suggesting significant potential for biofilm formation and biofouling events in hydraulic fracturing-produced water storage tank and other parts of the hydraulic fracturing infrastructure. More detailed analysis of this and other bacterial genomes recovered from hydraulic fracturing environments will lead to a better understanding of microbial activity in these environments.

### Accession number(s).

This whole-genome shotgun project has been deposited at DDBJ/ENA/GenBank under the accession number MCRW00000000. The version described in this paper is version MCRW01000000.
